# Editorial: Advances in the imaging techniques of radiologically subtle CNS disorders

**DOI:** 10.3389/fnins.2022.1059705

**Published:** 2022-11-03

**Authors:** Ahmad Raza Khan, Brian Hansen, Maryam Ardalan, Syed Shadab Raza

**Affiliations:** ^1^Centre of Bio-Medical Research (CBMR), Lucknow, India; ^2^Centre of Functionally Integrative Neuroscience, Aarhus University, Aarhus, Denmark; ^3^Department of Physiology, Institute of Neuroscience and Physiology, Sahlgrenska Academy, University of Gothenburg, Gothenburg, Sweden; ^4^Department of Stem Cell Biology and Regenerative Medicine, Era University, Lucknow, India

**Keywords:** neuroimaging, MRI, radiologically subtle, CNS, MRS, PET, CT

Most CNS disorders manifest with subtle structural, biochemical, or physiological alterations at an early stage of the disease. However, most early changes are not detectable with conventional neuroimaging modalities, rendering early diagnosis difficult. While this is valuable for understanding disease origin and development, for this reason, there is a strong need for the development of tools that are sensitive to radiologically subtle CNS alterations. Such tools would be invaluable for accurate, early diagnosis and for objective evaluation of treatment interventions. Therefore, this Research Topic focused on progress in the detection of radiologically subtle CNS disorders.

The title attracted 21 submissions from different parts of the world, such as China, Egypt, Finland, India, Japan, Saudi Arabia, Sweden, and the UK. A total of 12 articles were accepted of which 8 are research articles and 4 review articles. In total, the studies list contributions from 100 authors.

Here, we briefly summarize the articles published in this Research Topic. Algahtany et al. suggested that multimodal techniques, such as positron emission tomography (PET), PET-MRI, single-photon emission computed tomography, functional MRI (f-MRI), magnetic resonance spectroscopy (MRS), and diffusion tensor imaging (DTI), that can be employed in combination to detect a lesion, its connection, and its intimacy with eloquent areas to limit surgery and avoid post-surgical complications, particularly in epileptic patients. Likewise, Sone also suggested employing non-Gaussian diffusion MRI parameters and Arterial Spin Labeling (ASL) techniques can non-invasively detect microstructural parameters and hypo-perfusion, respectively, within the focused lesions. These advancements in technology may also provide usefulness for both focused detection and a better understanding of epilepsy. Quan et al. hypothesized that the radiomics features extracted from FLAIR and ADC images could be prognostic biomarkers for predicting clinical outcomes in acute ischemic stroke (AIS) patients. They developed a combined prediction model based on radiomics and DWI-ASPECTS and showed the superiority of the combined model. On retrospective data, the combined model showed unfavorable functional outcomes in patients with AIS. Zhu Q. et al. have shown that DKI parameters can significantly differentiate T1-hypointense lesions, T2-hyperintense lesions, normal-appearing white matter (WM), and WM microstructure in healthy controls. The damage in normal-appearing WM reflected by the DKI parameter, mean kurtosis could be a potential biomarker to evaluate the WM damage. This imaging-based classification in WM may help to evaluate the disease severity and progression. Jiang et al. performed a Tract-based Spatial Statistics (TBSS) study to explore WM integrity in the brain of End-Stage and Non-End-Stage Chronic Kidney Disease (ES-CKD and NES-CKD). The study showed that ES-CKD patients have more serious WM microstructure abnormalities than NES-CKD patients. The study also revealed that progressive WM abnormalities are associated with uric acid and phosphate levels in the blood. These findings also suggested that a multiparametric approach can provide a better understanding at a systemic level. Another study by Rawat et al. employed a multiparametric MRI approach that has revealed significant metabolic and volumetric alterations in the brain of patients with gluten ataxia (GA). *In-vivo* MRS revealed significant differences in NAA and NAA/choline ratios in the cerebellum. GA patients also had a significant reduction in total brain WM, cerebrum WM, and lateral ventricle and thalamic volume. Another study by Kumar et al. employed 1H MRS in two different brain regions viz., posterior parietal cortex and dorsolateral prefrontal cortex (DLPFC), to study neurochemical alterations in hyperthyroid patients, before and after antithyroid treatment. The 1H MRS has shown that hyperthyroidism causes changes in key metabolites in both regions, possibly indicating alterations in astrocyte physiology, glutamate/glutamine cycle, and/or oxidative stress. The altered neurometabolic ratios in the brain regions showed reversible changes after anti-thyroid treatment. The study strengthens the use of MRI in radiologically subtle CNS disorders and the outcome of efficient treatment intervention. Zhu L. et al. have used seed-based d mapping (SDM) a statistical technique for meta-analysis of neuroimaging studies. They studied hepatic encephalopathy (HE) patients for regional gray matter abnormalities using voxel-based morphometry. The study found gray matter volume reduction, especially in frontal regions, temporal insular cortex, and the caudate nucleus. The study also found an increase in plasma ammonia, which could be caused by increased blood flow to related brain regions in patients with cirrhosis. The study concludes that in patients with different degrees of HE, the changing area of gray matter volume was similar and the range of symptoms increased with aggravation. Zhu X. et al. explored whether the presence of impulse control disorder (ICD) in Parkinson's disease (PD) patients may determine abnormalities in the topological network by using rs-fMRI and graph theory methods. They divided PD patients into two groups, viz., PD-ICD and PD not ICD (PD-nICD) patients. The study showed that the clustering coefficient and characteristic path length of the brain functional network of PD-ICD patients increased, accompanied by damage to the Default Mode Network (DMN), Control Network (CN), and Dorsal Attention Network (DAN) nodes. The study also provides evidence for PD-ICD patients' brain network abnormalities from the perspective of information exchange. In another rs-MRI study, alterations in brain activity during microlesions in PD patients were investigated by Luo et al. They utilized the amplitude of low-frequency fluctuation (ALFF) and functional connectivity (FC) methods to explore changes associated with spontaneous brain activity and brain networks in PD patients before and after DBS surgery. The results demonstrated that implantation of DBS electrodes not only improves the activity of the basal ganglia-thalamocortical circuit but also reduces the activity of the DMN and ECN-related brain regions. These findings can be helpful for further understanding potential mechanisms that underlie microlesion in PD. Ai et al. have studied Rasmussen's Encephalitis (RE), a rare chronic neurological disorder characterized by unihemispheric brain atrophy and epileptic seizures. To reveal the involvement of genetic factors in the mechanisms of RE, whole-exome sequencing in 15 RE patients was performed in this study. They found single nucleotide variants (SNVs) in genes with the functions of antigen presentation, antiviral infection, epilepsy, schizophrenia, and nerve cell regeneration. The results suggest that RE is a polygenic disease and the triggering factors of the adaptive immune response may be SNVs related to antigen presentation and antiviral infection. Sasaguri et al. have reviewed the animal models of AD. The first-generation animal model expressed amyloid-beta (Aβ) peptide deposition and neuroinflammation. However, they have suffered from some artificial phenotypes, which could lead to erroneous interpretations of the outcome. The second-generation animal models of AD expressed Aβ pathology, neuroinflammation, vascular dysfunction as well as cognitive impairment in an age-dependent manner. However, these animal models also have some limitations. For instance, Aβ is resistant to proteolytic degradation and is therefore prone to aggregation in this model. The third-generation animal model exhibits more specific plaque pathology and neuroinflammation than the second-generation models and thus is more suitable for preclinical studies.

We strongly hope that this Research Topic will attract the attention of clinicians and researchers who are interested in developments toward a better understanding of CNS disorders where disease-related tissue alterations are too subtle to be radiologically visible at present. We thank not only all the authors for their precious contributions but also the reviewers for sharing their expertise. Their efforts have secured this Research Topic as a collection of high-quality articles, which can be useful to the clinical and research communities.

## Author contributions

AK wrote the editorial. BH, MA, and SR edited the editorial. All authors contributed to the article and approved the submitted version.

## Funding

AK got support from the Centre of Biomedical Research (CBMR), Government of Uttar Pradesh, and funded by Ramalingaswami Re-entry Fellowship-2019 of the Department of Biotechnology (DBT), Ministry of Science and Technology, Government of India. MA was supported by Lundbeck- an international postdoc fellowship. SR's lab is supported by a generous grant from the International Brain Research Organization.

## Conflict of interest

The authors declare that the research was conducted in the absence of any commercial or financial relationships that could be construed as a potential conflict of interest.

## Publisher's note

All claims expressed in this article are solely those of the authors and do not necessarily represent those of their affiliated organizations, or those of the publisher, the editors and the reviewers. Any product that may be evaluated in this article, or claim that may be made by its manufacturer, is not guaranteed or endorsed by the publisher.

